# *MolLM*: a unified language model for integrating biomedical text with 2D and 3D molecular representations

**DOI:** 10.1093/bioinformatics/btae260

**Published:** 2024-06-28

**Authors:** Xiangru Tang, Andrew Tran, Jeffrey Tan, Mark B Gerstein

**Affiliations:** Department of Computer Science, Yale University, New Haven, CT 06520, United States; Department of Computer Science, Yale University, New Haven, CT 06520, United States; Department of Computer Science, Yale University, New Haven, CT 06520, United States; Department of Computer Science, Yale University, New Haven, CT 06520, United States; Program in Computational Biology & Bioinformatics, Yale University, New Haven, CT 06520, United States; Department of Molecular Biophysics & Biochemistry, Yale University, New Haven, CT 06520, United States; Department of Statistics & Data Science, Yale University, New Haven, CT 06520, United States; Department of Biomedical Informatics & Data Science, Yale University, New Haven, CT 06520, United States

## Abstract

**Motivation:**

The current paradigm of deep learning models for the joint representation of molecules and text primarily relies on 1D or 2D molecular formats, neglecting significant 3D structural information that offers valuable physical insight. This narrow focus inhibits the models’ versatility and adaptability across a wide range of modalities. Conversely, the limited research focusing on explicit 3D representation tends to overlook textual data within the biomedical domain.

**Results:**

We present a unified pre-trained language model, MolLM, that concurrently captures 2D and 3D molecular information alongside biomedical text. MolLM consists of a text Transformer encoder and a molecular Transformer encoder, designed to encode both 2D and 3D molecular structures. To support MolLM’s self-supervised pre-training, we constructed 160K molecule-text pairings. Employing contrastive learning as a supervisory signal for learning, MolLM demonstrates robust molecular representation capabilities across four downstream tasks, including cross-modal molecule and text matching, property prediction, captioning, and text-prompted molecular editing. Through ablation, we demonstrate that the inclusion of explicit 3D representations improves performance in these downstream tasks.

**Availability and implementation:**

Our code, data, pre-trained model weights, and examples of using our model are all available at https://github.com/gersteinlab/MolLM. In particular, we provide Jupyter Notebooks offering step-by-step guidance on how to use MolLM to extract embeddings for both molecules and text.

## 1 Introduction

Pre-training techniques in natural language processing have resulted in significant shifts in numerous domains by enabling the interpretation of complex patterns from large-scale datasets (Radford *et al.* 2018, Vaswani *et al.* 2017, Devlin *et al.* 2019, Raffel *et al.* 2020, Wei *et al.* 2021). This success has led to promising outcomes in the biomedical field, particularly in tasks such as molecular property prediction and chemical structure generation, indicating strong potential for artificial intelligence in biomolecular studies (Gu *et al.* 2021, Xia *et al.* 2022, Luo *et al.* 2022a, Singhal *et al.* 2023).

Past endeavors have sought to learn representations of molecular chemical properties through supervised tasks using labeled data (Hwang *et al.* 2020, Zuranski *et al.* 2021). Nevertheless, these efforts heavily rely on the availability of large-scale datasets, prompting an increased focus on self-supervised learning. Recent investigations have leveraged pre-trained, self-supervised methods to derive representations of molecular structures (Zeng *et al.* 2022, Rong *et al.* 2020, Zhang *et al.* 2021), primarily employing a masking approach to learning the geometric structure and consequently obtaining corresponding chemical structure representations. However, these endeavors have predominantly concentrated on 2D molecular structures (Su *et al.* 2022, Pogány *et al.* 2018, Coley *et al.* 2017, Liu *et al.* 2023a), thereby neglecting the pivotal role of 3D structures in molecular modeling. In practical terms, 3D molecular structures encapsulate crucial chemical information, and the positions of functional groups within them serve as potent predictors for understanding molecular properties and modeling interactions. While some research has incorporated 3D geometric structures, these works have their limitations in model architecture (Yu *et al.* 2021, Miao *et al.* 2023, Jiang *et al.* 2021, Devinyak *et al.* 2014).

Additionally, the natural language modality encompasses a vast volume of biochemical knowledge, offering a valuable resource for enhancing model comprehension of molecular structures. When applied to downstream tasks, a model’s proficiency in interpreting natural language facilitates more refined control of molecule generation and editing, using natural language as the mode of interaction. Notably, recent state-of-the-art multimodal models for molecular tasks do not incorporate essential 3D information alongside natural language (Su *et al.* 2022). Hence, this underscores the imperative to develop models that can seamlessly integrate both 2D and 3D molecular structures with biomedical text to propel scientific discovery (Pei *et al.* 2024). Inspired by this, we introduce MolLM, the first molecular multimodal language model that combines 2D and 3D molecular structures with a natural language—a novel approach that has not been explored before.

In this work, we first create a multimodal dataset for self-supervised pre-training, including molecules sourced from PubChem (Kim *et al.* 2023) and related publicly available academic texts, totaling 160K molecule graph-text pairs. At the core of MolLM is a unified pre-training framework that seamlessly integrates a text Transformer encoder with a molecular Transformer encoder, pre-trained jointly on molecular graphs and related textual data. Specifically, the text Transformer encoder is pre-trained on biomedical text. To effectively capture the multimodal molecular structure information, we then adopt a molecular graph Transformer encoder. Leveraging the graph Transformer architecture, we integrate pairwise encodings to represent both 2D graphs and 3D geometric structures. In other words, we integrate positional encoding with the standard graph encoding, allowing the model to capture atom-wise structural information. Following Transformer-M (Luo *et al.* 2022b), we effectively leverage both 2D and 3D molecular structures to enhance performance in downstream tasks. Crucially, our model aligns these cross-modal representations during pre-training through the paradigm of contrastive learning. Contrastive learning efficiently facilitates self-supervised pre-training by bringing similar molecules closer together and placing dissimilar molecules farther apart in the representation space.

Our presented work further emphasizes the significance of capturing 3D molecular structures. MolLM overcomes the limitations of existing pre-trained molecular models, which can only process different data types separately, by providing a comprehensive understanding of molecular entities. We have conducted extensive experiments to showcase the model’s capacity to capture molecular expertise, demonstrating promising performance in downstream tasks such as (1) cross-modal retrieval (retrieving the related molecule or text with the query of the opposite modality), (2) molecule captioning (generating natural language descriptions of molecules), (3) property prediction (classifying molecule properties), and (4) molecule editing (editing molecules based on natural language prompts) (Zeng *et al.* 2022, Edwards *et al.* 2022, Wu *et al.* 2017, Liu *et al.* 2023b). Our promising results demonstrate that the benefits of integrating 3D information. For example, in the cross-modality matching task, while our multimodal architecture outperforms all baselines in various zero-shot and supervised settings, the direct incorporation of 3D data further improves each score. In summary, to the best of our knowledge, this is the first work that introduces a 2D and 3D pre-training framework for molecules in conjunction with textual information. We present two significant contributions to the field. First, we introduce a novel dataset, surpassing baseline datasets in size while maintaining comparable text complexity. Our results underscore the necessity of datasets containing profound textual information and substantial scale for maximizing effectiveness. Second, we demonstrate the efficacy of multimodal pre-trained models in molecular analysis, particularly when incorporating all three modalities of textual, 2D, and 3D information.

## 2 Related work

### 2.1 Molecular representations

Historically, many works have focused on 1D molecular representations, with the simplified molecular input line entry system (SMILES) being the most common method (Weininger 1988). Numerous papers have leveraged SMILES for a variety of molecular tasks (Chen and Zhang 2021, Kuenzi *et al.* 2020, An *et al.* 2022). For a more explicit representation of molecules, graph-based representations have become a popular method for integrating 2D structures. The most common and straightforward graph representation involves vertices representing atoms and their respective features, with edges representing bonds and their respective features. Utilizing the Open Graph Benchmark (Hu *et al.* 2020), various works have applied common graph model architectures for molecule analysis, such as CNNs, graph convolutional networks (GCNs), and GraphSAGE. (Pogány *et al.* 2018, Coley *et al.* 2017, Liu *et al.* 2023a). While these models achieve good results, they fail to account for nuances within the 3D molecular structure, such as stereochemistry, interaction energies, and spatial binding affinities. More recent works include 3D molecular graph data by providing 3D coordinates of each molecule within the atom representation. As 3D data are not universally available for many molecules, most studies generate 3D conformations using molecular analysis libraries such as RDKit (Landrum 2023). By focusing on 3D contexts, these models demonstrate improved performance on certain tasks where 3D structure significantly impacts outcomes, such as property prediction (Kuzminykh *et al.* 2018, Thomas *et al.* 2018, Kajita *et al.* 2017, Stärk *et al.* 2022, Li *et al.* 2022c, Yang *et al.* 2019). Some models use contrastive learning techniques to accommodate multiple modalities of molecule inputs, aiming to unify 2D and 3D structural information (Liu *et al.* 2022, Zhu *et al.* 2022, Li *et al.* 2022b).

### 2.2 Molecular pre-trained language models

The popularity of pre-trained language models has led to prior utilization of these models on molecules represented via textual formats such as SMILES. One of the initial models to explore inputting SMILES strings into language models is SMILES-Bidirectional Encoder Representations from Transformers (BERT) (Wang *et al.* 2019), which was trained on masking tasks to predict missing tokens in sequences representing text, including SMILES strings alongside regular text. Similarly, BARTSmiles expanded upon this approach by using a BART-like model and incorporating more downstream tasks (Chilingaryan *et al.* 2022). However, as these models use inherently 1D textual representations of molecules, they lose a significant amount of information regarding geometric and stereoelectric effects.

In terms of multimodality works, prior research has focused on multimodal text and molecule models to represent molecules and their related textual descriptions in a joint latent space. KV-PLM (Zeng *et al.* 2022) utilizes the SMILES representation of molecules before projecting them onto such a joint latent space, while MoMu (Su *et al.* 2022) utilizes a graph representation of molecules. These methods, along with other recent works (Wang *et al.* 2022a,b), utilize a contrastive loss function to align molecular representations and textual representations. To improve accuracy and robustness during training, MoMu employs augmentations of the molecular graphs to create semantically similar graphs (Liu *et al.* 2023b). However, due to both MoMu and KV-PLM’s molecular representation inputs, these models only consider a limited 2D view of molecular geometry.

Regarding training data, KV-PLM and MoMu each utilized datasets of 15K molecule-text pairings. KV-PLM sourced the textual data from PubChem, while MoMu sourced a different set of 15K pairs from the S2ORC (Lo *et al.* 2020) corpus dataset. As the S2ORC dataset lacks clear pairing between molecules and related text, Su *et al.* expressed concerns that simply selecting texts referencing molecules might not guarantee strongly related text. Therefore, limitations in the datasets of prior works extend beyond concerns about their size, as these datasets may also exhibit weak correlations within their molecule and text pairings.

## 3 Materials and methods

### 3.1 Data

To facilitate self-supervised pre-training of molecules, a large-scale text-molecule paired dataset was necessary. Consequently, we collected our dataset from two sources. First, for collecting the molecular data and directly related descriptions, we accessed the PubChem molecular database (Kim *et al.* 2023), which hosts information on over 100 million molecular compounds. From this repository, we used the PubChem Classification Browser to identify 160K molecules with substantial physical descriptions. Specifically, our criterion was to select entries with at least 150 characters in the “Record Description” field. For example, we utilized the PubChem Classification Browser to acquire molecules such as “acetylcarnitine.” Then, using the PubChem REST API, we queried for 2D graphical data, as well as the basic physical descriptions available from PubChem.

To collect associated textual data, we followed the matching methodology employed by MoMu to extract sentences from papers within the S2ORC corpus database (Lo *et al.* 2020) relevant to certain molecules. We then paired these text descriptions with the 2D and 3D graphical representations of each molecule to create molecular graph-text pairs. Specifically, using the aforementioned example, we searched the S2ORC database to find papers related to “acetylcarnitine,” extracting text containing the molecule or its synonyms from the Abstract, Introduction, and Conclusion sections of each paper. Due to the length limit of the text encoder, we constructed our textual data with a cut-off limit of 256 tokens.

Notably, while MoleculeSTM (Liu *et al.* 2023b) has a large dataset with both 2D and 3D data, it does not incorporate academic textual data, thus overlooking a significant data source. Table 1 provides a comparison of our dataset against similar datasets. Our dataset is the largest within this domain, encompassing academic text and comprehensive multimodal molecule information.

**Table 1:** Comparison of datasets used in our study (MolLM) and the baseline approaches in this field.^a^

Model	Input	Text for pretraining	2D	3D	# Molecules	Training source
MoMu (Su *et al.* 2022)	SMILES	✓	✓	✗	15K	PubChem, S2ORC
MoleculeSTM (Liu *et al.* 2023b)	PubChem Descriptions	✗	✓	✓	281K	PubChem
KV-PLM (Zeng *et al.* 2022)	SMILES	✓	✓	✗	10K	PubChem, S2ORC
MolLM	SMILES + PubChem Descriptions	✓	✓	✓	160K	PubChem, S2ORC

a Each dataset is characterized by (columns 2–7) input data type, availability of text pretraining, availability of 2D/3D structural information pretraining, total molecule count, and the pre-trained data source.

### 3.2 Architecture

**Figure 1.**
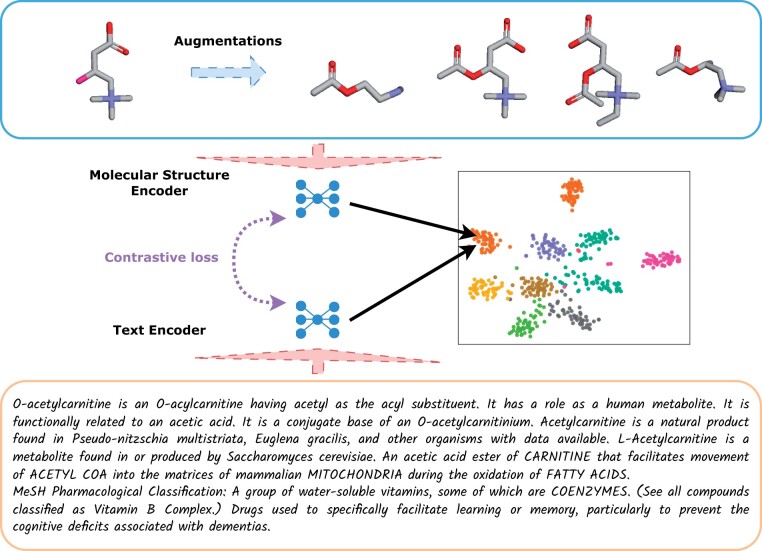
Overview of the pretraining pipeline. MolLM encodes both molecular graph data (top) and textual data (bottom). For each molecule, we generate up to four molecule augmentations before encoding the 2D and 3D structural data. On the text side, we encode related sentences from scientific journals. These encoded representations are then used during the pretraining for contrastive learning.

To facilitate the encoding of both molecules and text into MolLM’s joint latent space, we employ a text encoder for the textual descriptions of molecules and, separately, a molecule encoder for the 2D/3D representations of molecules, as shown in Fig. 1.

#### 3.2.1 Text encoder

We initialize our text encoder’s model weights with KV-PLM’s checkpoint (Zeng *et al.* 2022), aiming to generate embeddings for the extracted sentences from academic literature in our dataset. KV-PLM enhances BERT (Devlin *et al.* 2019), a widely used general text encoder, by fine-tuning its performance on academic texts related to molecules, including SMILES strings (Weininger 1988).

We also double the tokenizer output length limit within KV-PLM’s use of the BERT tokenizer, increasing it from 128 tokens to 256 tokens. This adjustment helps avoid unnecessary truncation and loss of detail when processing text sequences, allowing for the inclusion of more detailed text descriptions. Thus, our approach enables the efficient capture of the core meaning and context of full sentences and paragraphs.

#### 3.2.2 Molecular structure encoder

MolLM is designed to obtain representations of molecular data via pathways that process both 2D and 3D structural information. The 2D pathway harnesses information extracted from the molecule’s 2D graph structure, including degrees, shortest path distances, and edges. These data elements represent the spatial relationships among atoms in the molecule, enabling the model to understand its 2D architecture. By contrast, the 3D pathway focuses on the molecule’s 3D geometrical structure, computing spatial distances among atoms within the 3D structure. This geometric perspective empowers the model to gain insights into the 3D arrangement of atoms and their complex interactions, which are often crucial in predicting molecular properties and interactions. Together, these three pathways equip MolLM with a thorough comprehension of the molecule, spanning its sequence and both its 2D and 3D structures, thereby producing a rich, multimodal representation.

Inspired by Transformer-M (Luo *et al.* 2022b), MolLM employs a similar strategy to process both 2D graph structures and 3D geometric structures in molecular representations within a unified model. Using a graph Transformer as its base, MolLM encodes structural data such as edge features, bond type, and 3D spatial relation as bias values within the attention mechanism. The Transformer uses these values to weigh and combine different parts of the input. We implement this modification in the attention mechanism because it allows the relationship between atoms in the model to be directly influenced by their 2D and 3D spatial relations, aligning with the physical significance of these geometric relations.

To summarize the implementation, we recap the encodings of these structural features that we adopt from Transformer-M. In terms of the graph structure, vertices represent atoms and edges represent the bonds between these atoms.

For encoding edge features, we utilize the 2D graph structure by considering edge feature row vectors, $e_{1},e_{2},\ldots$, along the shortest path between any two vertices. These edge feature vectors represent characteristics of the chemical bonds, including bond type and stereochemistry. Suppose the edge feature vectors are of dimension *k*_edge_, as in $e_{i}\in R^{k_{\mathrm{edge}}}$ for each edge. Then, the element-wise average of them is denoted as avg(e). We also utilize a learnable vector $w\in R^{k_{\mathrm{edge}}}$ to emphasize the importance of certain bond features over others. Our encoding of edge features for any two vertices indexed as *i* and *j*, which is a scalar quantity, is given as

(1) $$\phi_{ij}^{\text{Edge}}=\mathrm{avg}(e)\cdot w.$$

Additionally, we aim to include 2D distance, specifically the shortest path between any two atoms by following their bonds, in our structural encoding. Thus, for the relationship between atoms in the 2D space, we define an encoding to represent the shortest path distance between two atoms. Moreover, we introduce a learnable scalar *w* to allow for weighing the importance of considering shortest path distances in our structural encoding. We define *spd*(*i*, *j*) as the shortest path distance between atoms *i* and *j* in the 2D graph, counted by edges, making it a scalar quantity. Hence, our encoding for shortest path distances, also a scalar quantity, is

(2) $$\phi_{ij}^{\text{SPD}}=\mathrm{spd}(i,j)\cdot w.$$

Similarly, we consider 3D spatial relations. Instead of utilizing standard Euclidean distance, we employ the Gaussian Basis Kernel as it provides a non-linear function of distance, offering a more nuanced representation of the 3D spatial relationship. Suppose we utilize *K* Gaussian Basis Kernels to represent 3D distance with the vector $\psi (i,j)=[\psi (i,j,1),\ldots ,\psi (i,j,K)]\in R^{K}$. We also introduce a learnable matrix $W_{1}\in R^{K\;\times \;K}$ and a vector $w_{2}\in R^{K}$ for a multi-layer perceptron (MLP) to obtain the final encoding by processing the distance representation given by the Gaussian Basis Kernel. The MLP allows for a richer representation with higher-level features that can be inferred from the 3D distance along with the ability to selectively emphasize certain aspects, such as specific directions within the 3D distance. We utilize ReLU as the activation function, *act*, within this MLP. The 3D distance encoding is processed between vertices, indexed as *i* and *j*, as a scalar quantity, after applying the Gaussian Basis Kernel as

(3) $$\phi_{ij}^{3D\;\text{Distance}}=\mathrm{act}(\psi (i,j)\;\times \;W_{1})\cdot w_{2}.$$

Specifically, the Gaussian Basis Kernel has a hyperparameter *σ* that controls the width of the kernel, which adjusts the sensitivity of the kernel to distance and the smoothness of its output. These aspects of the kernel align with the scales of distances at the molecular level and the potential noise of these distances, respectively. The *k*-th Gaussian Basis Kernel (Scholkopf *et al.* 1997) within ψ(i,j) is given as

(4) $$\psi (i,j,k)=\text{exp}\;\left(-\frac{||x_{i}-x_{j}|{|}^{k}}{\sigma^{k}}\right).$$

These 2D and 3D positional encodings from the graph structure of the molecule and the 3D geometric structures are linearly combined to consider them in aggregate. We incorporate this combined encoding into the attention mechanism of the graph Transformer layers. This linear combination, considering all atoms in the molecular graph, is given as

(5) $$\phi_{ij}^{2D/3D\;\text{Position}}=\phi_{ij}^{\text{Edge}}\;+\;\phi_{ij}^{\text{SPD}}\;+\;\phi_{ij}^{3D\;\text{Distance}}.$$

Let *a* be the atom count in the molecular graph. We define the matrix $\phi^{2D/3D\;\text{Position}}\in R^{a\;\times \;a}$ by Equation (5). We directly add this matrix as a bias to the standard Transformer attention calculation before the final application of Softmax. The motivation behind adding this matrix to the Transformer attention mechanism is to capture contextual relationships among our graph’s vertices and atoms while weighing the importance of each in the final embedding. By incorporating our bias, which is based upon 2D and 3D spatial data, the model directly considers this rich spatial data in the attention calculation, thus accounting for spatial relationships between atoms within the molecular structure.

### 3.3 Pre-training

For MolLM’s pretraining, data augmentation strategies allow us to enhance robustness and efficiency in handling molecular-related tasks, as demonstrated in (Su *et al.* 2022). We utilize contrastive learning as the objective, aiming to derive meaningful representations by comparing pairs of positive and negative samples. Our approach involves two types of losses: (1) *a contrastive loss*, which compares representations of different modalities within the same data sample and (2) *a self-loss*, which contrasts different augmentations of the same modality within the same data sample. We define these two losses in Section 3.3.2.

#### 3.3.1 Data augmentation

We expand upon the data augmentations proposed in MoMu (Su *et al.* 2022) by introducing two augmentations that alter each molecule’s chemical features, such as molecular motifs and functional groups. These additions complement the two augmentations used in MoMu, which alter graph features like nodes and edges. In total, we apply four data augmentations to each 2D molecular graph: (1) *node dropping*, (2) *subgraph sampling*, (3) *chemical transformation*, and (4) *substructure removal*. We then compute and insert 3D atomic positions into these augmented graphs, aiming to construct semantically consistent molecular structures suitable for pre-training.

*Node dropping* randomly discards vertices from the original graph, while *subgraph sampling* involves traversing random walks. *Chemical transformation* augmentations generate new structures by applying various chemical transformations alongside other graph augmentations. These transformations entail adding and removing functional groups, followed by the optimization of 3D structures. *Substructure removal* uses BRICS decomposition to remove certain substructures in a molecularly viable manner. From the possible substructure generations, a random viable molecular motif is chosen. Figure 1 provides a visualization of the augmentations, and Supplementary appendix S3 shows more examples. Even with some augmentations modifying the chemical structure and properties, we consider the new instances to belong to the same label. Recent work has demonstrated the effectiveness of such augmentations during pre-training, improving model robustness, generalization, and understanding of functional groups and chemical structure compositions.

For the aforementioned augmentations, we perform manipulations and optimizations on molecular structures using the open-source RDKit library. These augmentations involve a deliberate randomization process and are repeated until we obtain an augmented molecule that measures a difference below 3.0 root-mean-square deviation (RMSD) according to RDKit’s molecular distance, or inverse similarity, calculation. We chose this threshold of 3.0 based on expert annotation and discussions with two chemistry professors, after examining the similarity of 100 augmented molecules for various RMSD values. This criterion helps avoid augmenting molecules to be too semantically different, with too large of a change in molecular properties, from the original. See Supplementary appendix S2 for further motivation behind our use of RDKit. Furthermore, for each molecule within the training dataset, only the four augmented versions are input into training, while the original molecule is not. There may be a concern with not using the original molecule, but our proportion of atoms to drop for *node dropping* is a conservative 10%.

Given a mini-batch of *N* molecular graphs, we generate four different augmentations for each graph. Let $z_{i}^{G_{1}},\;z_{i}^{G_{2}},\;z_{i}^{G_{3}}$, and $z_{i}^{G_{4}}$ denote the representations of the augmented versions of the *i*th graph $G_{i}$. Let $z_{i}^{T_{1}},\;z_{i}^{T_{2}}$, and $z_{i}^{T_{3}}$ denote the representations of three different sentences describing the *i*-th graph $G_{i}$. The objective of contrastive learning is to minimize the contrastive loss for each graph in the mini-batch, considering both cross-modal and self-contrastive losses.

#### 3.3.2 Contrastive learning objective

The purpose of the cross-modality contrastive loss is to align representations of molecules and text in the latent space, while the self-loss aims to align augmented representations within the same modality. We define the cross-modality contrastive loss as $L_{\text{cross}}$ and self-contrastive loss as $L_{\text{self}}$ for the *i*-th graph within a mini-batch of *N* molecular graphs, each with three different augmentations. The final contrastive learning objective combines cross-modal and self-contrastive losses: $L_{\text{MolLM}}=L_{\text{cross}}\;+\;L_{\text{self}}$, where $\ell_{i}^{\left(\cdot \right)}$ calculates the contrastive loss for the *i*-th graph $G_{i}$ considering the respective pairs of multimodal representations.

**Figure 2.**
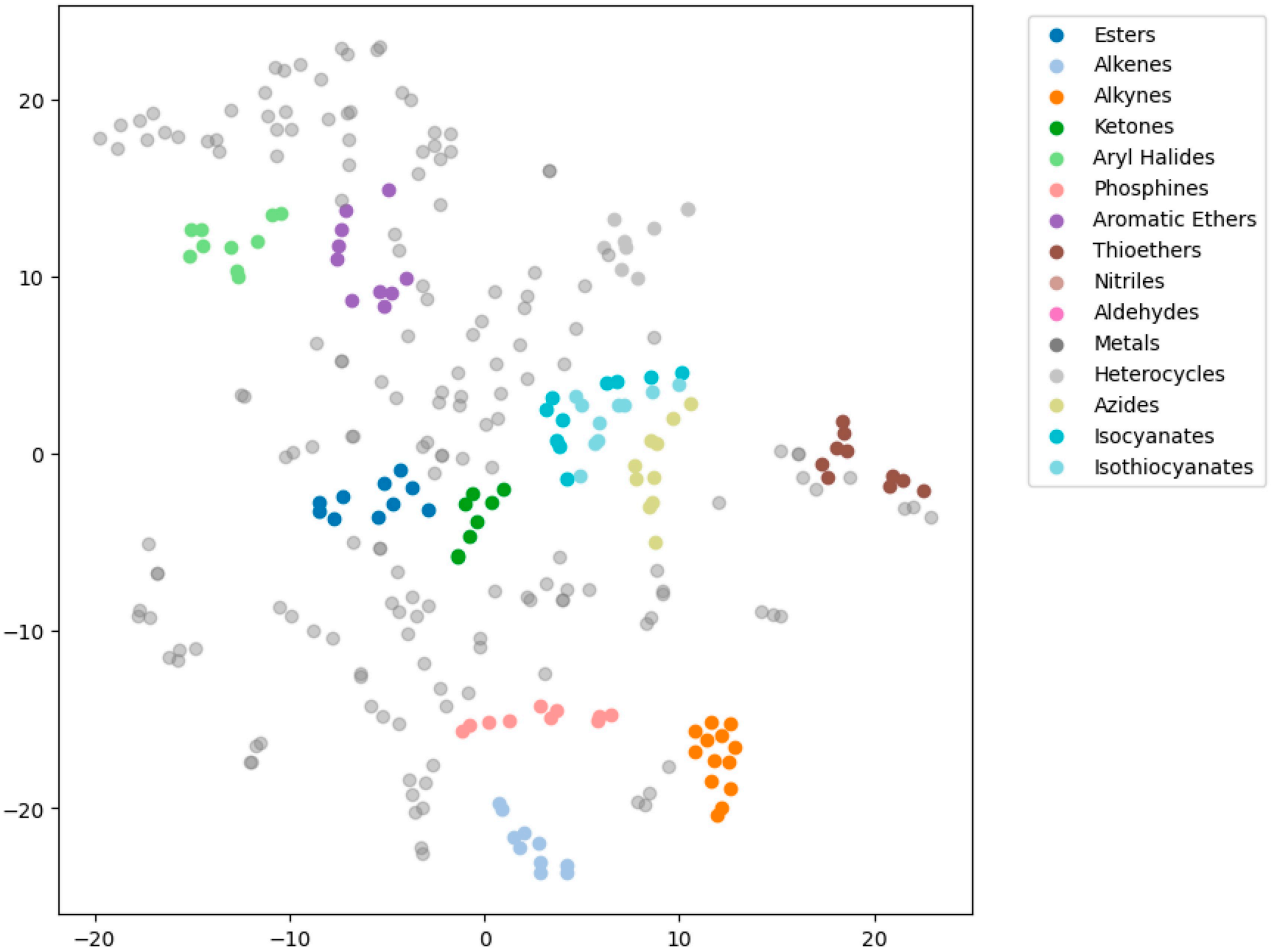
t-SNE visualization of molecule embeddings in MolLM’s joint latent space for select groups of molecules. Colored points represent molecules belonging to the select groups while the gray points represent a variety of other molecules. We displayed 200 data points for a clearer visualization and procured assistance from a chemistry expert to perform annotations of categorization.

For an explanation of contrastive loss, $L_{\text{cross}}$, recall that for the *i*-th graph of *N* molecular graphs, $z_{i}^{G_{1}},\;z_{i}^{G_{2}},\;z_{i}^{G_{3}}$, and $z_{i}^{G_{4}}$, are four representations of augmented graphs, and $z_{i}^{T_{1}},\;z_{i}^{T_{2}}$, and $z_{i}^{T_{3}}$ are representations of related text. Equation (6) describes the contrastive loss as a measure of similarity between the graph representations ($z_{i}^{G_{a}}$) and the corresponding text representations ($z_{i}^{T}$) for a given molecular graph. This loss function is formulated as a negative logarithm of the ratio of the exponential similarity between the matching graph and text representations (for a specific graph-text pair) to the sum of exponential similarities across all possible graph-text pairs within the batch of *N* molecular graphs. The term $\mathrm{sim}(z_{i}^{G_{a}},z_{i}^{T})$ denotes the cosine similarity function between a graph representation and a text representation, and *τ* is a temperature parameter that scales the similarity scores. The loss aims to maximize the numerator, which represents the similarity between matching pairs, and minimize the denominator, which represents the similarity between non-matching pairs. This approach encourages the model to produce graph and text representations that are closer in the embedding space for matching pairs while pushing non-matching pairs farther apart. Figure 2 displays a t-SNE visualization of molecule embeddings generated by MolLM, illustrating distinct clusterings for each molecule type. This demonstrates the model’s capability to characterize molecules via rich representations.

(6) $$\ell_{\mathrm{cros}s_{i}}=-\text{log}\;\frac{\;\text{exp}(\mathrm{sim}(z_{i}^{G_{a}},z_{i}^{T})/\tau )}{\sum_{j=1}^{N}\;\text{exp}\;(\mathrm{sim}(z_{i}^{G_{a}},z_{j}^{T})/\tau )},$$

Similarly, for the self-loss $L_{\text{self}}$, recall the same notation for the augmented graphs and related text. This loss function in Equation (7) is defined as the negative logarithm between the ratio of the sum of exponential similarities between all pairs of augmented graph representations ($z_{i}^{G_{a}},z_{i}^{G_{b}}$) to the sum of all exponential similarities of all such pairs in the graph. The effect of this loss is to ensure that different augmented representations of the same molecular graph are similar to each other in the embedding space. The parameter *τ* is a temperature parameter that scales the similarity scores. This loss encourages the model to push different augmented views of the same graph closer together while pushing those of different graphs farther apart.

(7) $$\ell_{\mathrm{sel}f_{i}}=-\text{log}\;\frac{\sum_{G_{a},G_{b}\in \left(\begin{array}{c} \left\{G_{1},G_{2},G_{3},G_{4}\right\}\\ 2 \end{array}\right)}\;\text{exp}\;(\mathrm{sim}(z_{i}^{G_{a}},z_{i}^{G_{b}})/\tau )}{\sum_{j=1}^{N}\sum_{G_{a},G_{b}\in \left(\begin{array}{c} \left\{G_{1},G_{2},G_{3},G_{4}\right\}\\ 2 \end{array}\right)}\;\text{exp}\;(\mathrm{sim}(z_{i}^{G_{a}},z_{j}^{G_{b}})/\tau )},$$

Here, the notation $\left(\begin{array}{c} \left\{G_{1},G_{2},G_{3},G_{4}\right\}\\ 2 \end{array}\right)$ represents the selection of any combination of two different graphs from the set of four. These four graphs represent the molecules modified by four different augmentations.

(8) $$L_{\text{cross}}=\frac{1}{N}\sum_{i=1}^{N}\ell_{\mathrm{cros}s_{i}}^{\left(z_{i}^{G_{1}},z_{i}^{G_{2}},z_{i}^{G_{3}},z_{i}^{G_{4}},z_{i}^{T_{1}},z_{i}^{T_{2}},z_{i}^{T_{3}}\right)}$$

 

(9) $$L_{\text{self}}=\frac{1}{N}\sum_{i=1}^{N}\ell_{\mathrm{sel}f_{i}}^{\left(z_{i}^{G_{1}},z_{i}^{G_{2}},z_{i}^{G_{3}},z_{i}^{G_{4}}\right)}$$

In brief, the contrastive learning objective consists of two components: cross-modal contrastive loss and self-contrastive loss. The cross-modality contrastive loss minimizes the distance between the different modalities (i.e. molecular graphs and related textual descriptions) of the same molecule while maximizing the distance between different molecules. The self-contrastive loss minimizes the distance between different augmentations of the same molecule while maximizing the distance between augmentations of different molecules. Thus, the model learns to generate more robust and semantically meaningful representations for both molecules and text in a joint latent space.

### 3.4 Fine-tuning downstream tasks

To showcase the adaptability and practicality of our model across a range of downstream tasks that necessitate both molecular and textual inputs, we fine-tune our model on four such tasks. Cross-modality matching involves retrieving the correct molecular graph or text from a list of data in the opposing modality. Property Prediction entails classifying various experimental molecular properties, many related to biological activities such as blood–brain barrier penetration. Molecule Captioning generates a useful text description of a molecule, while molecule editing generates a new molecule given an original molecule and a text prompt with a desired property. See Table 2 for a summary of these tasks.

**Table 2.** Description of downstream tasks tested along with their associated dataset source (database or paper providing this data) and modalities.

Task	Definition	Dataset	Modalities
Cross-modality matching	Given a key molecule or text, find the associated item of the opposite modality.	PCdes	Text, 2D, 3D
Property prediction	Given a molecule, predict properties with classification tasks.	MoleculeNet	2D, 3D
Molecule caption	Given a molecule, generate a descriptive text.	MolT5	Text, 2D, 3D
Molecule editing	Given a molecule and a text prompt, generate an edited molecule accordingly.	MoleculeSTM	Text, 2D, 3D

#### 3.4.1 Cross-modality matching

**Figure 3.**
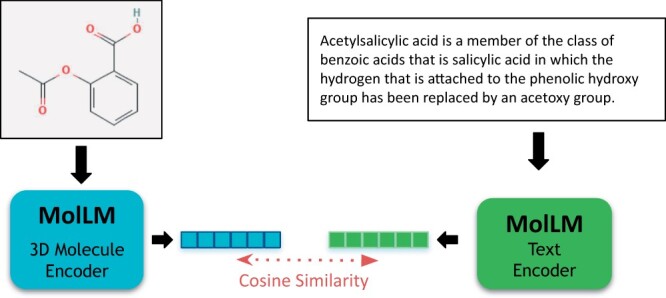
Visualization of the cross-modality matching text for a given pair of molecules and text. Embeddings are generated for each molecule and text using the respective encoders of MolLM. Then, cosine similarity is used to find the most similar pair for top-1 matching, or the 20 most similar for R@20 (Recall), for the matching task.

To implement the cross-modality matching task using our model, we begin by using the two encoders to produce embeddings for all of the text and molecules, respectively. A “key” text or molecule is used as a query to find a match in the opposing modality. We generate embeddings for the “key” text or molecule, as well as for the set of texts or molecules that will be used for querying. We then compare the “key” text or molecule against the set of the opposite modality by selecting those with maximal cosine similarity between their embeddings, aiming to find the best match (or matches, in the context of the recall metric). See Fig. 3 for a visualization of how the model considers a specific molecule and text pair by computing cosine similarity for the task. For fine-tuning this task, similar to (Su *et al.* 2022), we split the data into a training set of 10 500 molecules, a validation set of 1500 molecules, and a test set of 3000 molecules, while generating their 2D graphs.

#### 3.4.2 Property prediction

For the property prediction task, a classification task, we utilize our model by employing only the 2D/3D molecular encoder to produce an embedding for the molecule. Then, we attach a graph prediction head to our molecular encoder, yielding a 768-dimensional molecule embedding, as described in Supplementary appendix S1. Additionally, we explore the use of a random forest classifier instead of this fully connected prediction head.

#### 3.4.3 Molecule captioning

**Figure 4.**
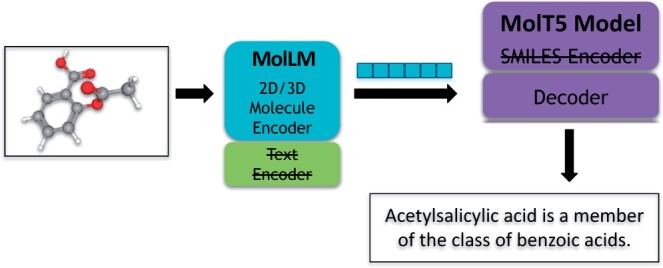
Visualization of the molecule captioning task. We begin with a 2D/3D molecular graph of acetylsalicylic acid, which is then encoded through MolLM’s molecule encoder. This encoded representation is then decoded through MolT5’s decoder, which produces a molecule-specific caption.

For the implementation of the Molecule Captioning task, we utilize the 2D/3D molecular encoder of our model to produce an embedding for the molecules. Subsequently, we forward this embedding through the decoder of the MolT5 model (Edwards *et al.* 2022) to generate the descriptions, as our model lacks a decoder for text. We utilize both the small (∼77M parameters) and base (∼ 250M parameters) checkpoints of MolT5. Refer to Fig. 4 for a visualization of this technique for generating captions. The -small and -base suffixes on the model names denote the checkpoint of MolT5 used.

#### 3.4.4 Molecule editing

**Figure 5.**
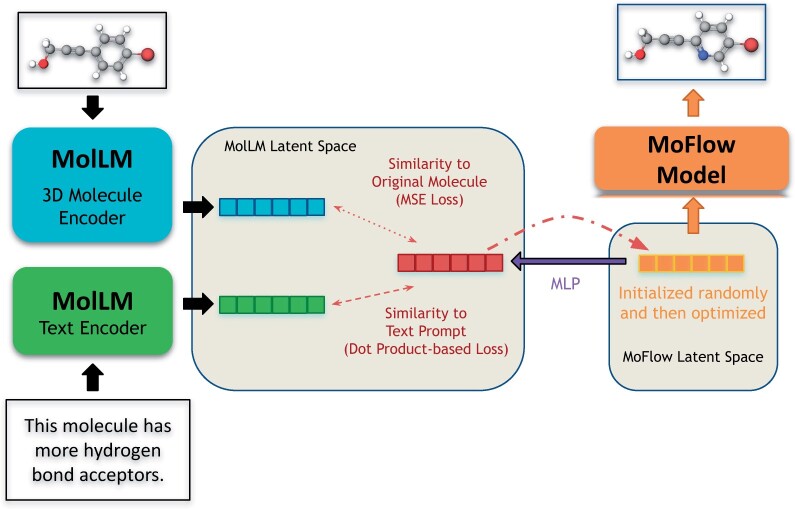
Visualization of molecule editing task. We start with a molecular graph and an instruction on how to edit the molecule. The graph and text are sent to MolLM’s molecule and text encoders, respectively. We optimize an embedding in MoFlow’s latent space but with two losses, to retain similarity to the original molecule while following the text prompt, computed on a translated embedding within MolLM’s latent space. The optimized embedding within MoFlow’s latent space is then reversed through MoFlow to obtain the final molecule.

Finally, for the molecule editing task, we utilize an input molecule and a text prompt. The desired output is a generated molecule that retains similarity to the input molecule while having properties indicated in the text prompt. As MolLM does not have a decoder to directly reverse its embeddings into molecular graphs, we utilize MoFlow (Zang and Wang 2020), an invertible flow-based molecular model, for generation. We optimize the embedding in MoFlow’s latent space to enable its reversal into the final edited molecule, while computing losses within MolLM’s latent space. Refer to Fig. 5 for a diagram of this process. We pass the input molecule and text prompt through the molecule and text encoders of MolLM, respectively, to obtain embeddings for each. In the diagram, these are represented as blue and green rectangles, respectively.

To begin the optimization process, we initialize an embedding in the MoFlow latent space, represented by the orange rectangle in the diagram, with a random embedding from Gaussian noise. Then, we translate this embedding into an embedding within MolLM’s latent space, in which we denote the intermediate embedding by an MLP, represented by the purple arrow in Fig. 5. Refer to Supplementary Appendix S4 for details of this MLP. Next, to indirectly optimize the embedding within the MoFlow latent space, we compute two losses within the MolLM latent space (as shown in Fig. 5 by double-headed red dashed arrows). The first loss aims to maintain similarity to the embedding of the original molecule. The second loss aims to align the embedding of the original molecule with the embedding of the text prompt to achieve the desired molecular properties. See Supplementary Appendix S3 for details of these losses. After the losses within the MolLM latent space are computed, the gradients with respect to the embedding within the MoFlow latent space, represented by the orange in the diagram, are backpropagated, as shown by a curved red arrow. Then, these gradients are used to optimize the embedding in MoFlow latent space to minimize both of the aforementioned losses. Thus, the optimized embedding represents a molecule with similarity to the input molecule but possessing the properties described in the text prompt. After 600 optimization steps, we obtain the final state of the embedding. Ultimately, we reverse the embedding, represented by orange in Fig. 5, through the invertible MoFlow model to produce the final edited molecule, as illustrated by the orange arrow.

## 4 Results

### 4.1 Cross-modality matching

**M-T** (molecule-to-text) retrieval begins with a 3D molecular graph and aims to select the most relevant textual descriptions associated with the given molecule. Conversely, **T-M** (text-to-molecule) retrieval starts with a text description and attempts to identify the molecular graph that best corresponds to the description. Both supervised tasks utilize the PCDes dataset (Zeng *et al.* 2022), which comprises SMILES strings and their corresponding textual descriptions. The dataset used for zero-shot learning is consistent with the one composed by MoMu. We generate 2D graphs for each example and then convert these SMILES strings into PubChem CIDs. To incorporate 3D conformation data into our model, we modify each graph by adding 3D information using RDKit’s MMFF94 implementation. Similar to (Zeng *et al.* 2022, Su *et al.* 2022), we perform retrieval in randomly selected batches of 64, assessing the average accuracy of top-1 retrieval results and the recall of top-20 (R@20) retrieval results. Specifically, the recall of top-1 retrieval measures how often the model retrieves the correct result as its best match, while the recall of top-20 retrieval measures how often the best match is among what the model deems to be the top 20 most similar results. For each task, we create both sentence-level and paragraph-level tasks. In the sentence-level setting, following (Zeng *et al.* 2022, Su *et al.* 2022), we use a randomly chosen sentence from the molecule’s description. In the paragraph-level setting, we utilize the entire description. We adopt Sci-BERT, KV-PLM, and MoMu as baselines.

From Table 3, it is clear that MolLM-3D achieves notable results by outperforming all baselines across each task. This performance is evident in both zero-shot and supervised settings, encompassing sentence-level and paragraph-level tasks. Particularly impressive is the M-T R@20 (Recall) score of 92.05 ± 1.03 in the supervised sentence-level task for MolLM-3D, indicating a high recall rate and suggesting that the model is highly effective in retrieving relevant textual descriptions for given molecular structures. Similarly, T-M accuracy and recall at both the sentence level and paragraph level in the supervised setting show a marked improvement over the other models, with MolLM-3D scoring 66.11 and 91.75, respectively. As MolLM-3D consistently outperforms MolLM-2D, these results demonstrate the efficacy of incorporating 3D structural information into the retrieval process.

**Table 3.** Molecule-to-text (M-T) and text-to-molecule (T-M) retrieval results, presented across four different settings: sentence-level zero-shot prediction, sentence-level supervised fine-tuning, paragraph-level zero-shot prediction, and paragraph-level supervised fine-tuning.^a^

Model	M-T Acc	M-T R@20	T-M Acc	T-M R@20
Sentence-level and zero-shot				
Sci-BERT	$1.38^{\pm 0.09}$	$0.38^{\pm 0.06}$	$1.57^{\pm 0.05}$	$0.28^{\pm 0.03}$
KV-PLM	$1.42^{\pm 0.13}$	$0.64^{\pm 0.35}$	$1.43^{\pm 0.06}$	$0.31^{\pm 0.08}$
MoMu	$39.07^{\pm 1.11}$	$37.36^{\pm 0.83}$	$38.51^{\pm 1.04}$	$31.18^{\pm 1.04}$
MolLM-2D	$45.46^{\pm 0.99}$	$46.60^{\pm 0.81}$	$45.25^{\pm 0.90}$	$41.17^{\pm 0.92}$
MolLM-3D	**45.82** ^±0.87^	**47.29** ^±0.85^	**46.02** ^±0.90^	**41.99** ^±0.94^
Sentence-level and supervised				
Sci-BERT	$50.39^{\pm 1.39}$	$62.11^{\pm 1.39}$	$50.12^{\pm 1.67}$	$68.02^{\pm 1.87}$
KV-PLM	$53.79^{\pm 1.42}$	$66.63^{\pm 1.51}$	$55.22^{\pm 0.94}$	$71.80^{\pm 1.56}$
MoMu	$58.74^{\pm 1.61}$	$81.29^{\pm 0.99}$	$54.94^{\pm 0.73}$	$78.29^{\pm 0.56}$
MolLM-2D	$64.59^{\pm 1.20}$	$91.73^{\pm 2.03}$	$64.83^{\pm 1.89}$	$91.20^{\pm 1.82}$
MolLM-3D	**66.11** ^±0.84^	**92.05** ^±1.03^	**66.10** ^±1.05^	**91.88** ^±1.79^
Paragraph-level and zero-shot				
Sci-BERT	$1.38^{\pm 0.01}$	$0.34^{\pm 0.05}$	$1.56^{\pm 0.05}$	$0.32^{\pm 0.03}$
KV-PLM	$1.94^{\pm 0.04}$	$0.40^{\pm 0.03}$	$1.82^{\pm 0.12}$	$0.52^{\pm 0.05}$
MoMu	$46.66^{\pm 0.31}$	$43.66^{\pm 0.28}$	$45.66^{\pm 0.12}$	$43.52^{\pm 0.14}$
MolLM-2D	$54.81^{\pm 0.82}$	$54.24^{\pm 0.87}$	$54.95^{\pm 0.85}$	$54.64^{\pm 0.90}$
MolLM-3D	**55.90** ^±0.88^	**55.44** ^±0.80^	**55.06** ^±0.83^	**54.88** ^±0.86^
Paragraph-level and supervised				
Sci-BERT	$62.57^{\pm 1.33}$	$60.67^{\pm 0.21}$	$61.75^{\pm 0.74}$	$60.77^{\pm 0.68}$
KV-PLM	$64.81^{\pm 0.81}$	$63.87^{\pm 1.14}$	$64.95^{\pm 1.47}$	$64.27^{\pm 1.05}$
MoMu	$81.09^{\pm 0.21}$	$80.15^{\pm 1.06}$	$81.45^{\pm 0.51}$	$80.62^{\pm 0.44}$
MolLM-2D	$86.33^{\pm 1.80}$	$90.67^{\pm 2.11}$	$87.00^{\pm 1.83}$	$91.06^{\pm 1.95}$
MolLM-3D	**87.05** ^±1.94^	**90.98** ^±1.79^	**87.85** ^±1.97^	**91.75** ^±1.26^

a R@20: top 20 recall. The scale of the accuracy and recall values is consistent with Zeng *et al.* (2022). "Bold value" refers to the best performance.

### 4.2 Property prediction

Property prediction is a downstream task widely used in prior work, such as (Su *et al.* 2022) and (Liu *et al.* 2023b). Similar to these studies, we utilize the MoleculeNet (Wu *et al.* 2017) benchmark, aimed at assessing how effectively a pre-trained molecular graph encoder can adapt to various disparate classification tasks. For example, the TOX21 and HIV datasets might prompt a model to classify new molecular graphs for toxicity markers or anti-HIV activity, respectively. We include baselines such as random forest (Zhu *et al.* 2021); RXNFP, BERT, and SMI-BERT (Zeng *et al.* 2022); GCN (Kipf and Welling 2016); GIN (Xu *et al.* 2018); KPGT (Li *et al.* 2022a); KANO (Fang *et al.* 2023); and MoLFormer-XL (Ross *et al.* 2022). Refer to Supplementary appendix S8 for details on these baselines. However, we only directly compare our model with relevant approaches, including MoMu (Su *et al.* 2022) and KV-PLM (Zeng *et al.* 2022), that utilize a similar text and molecule multimodal strategy as our model. As shown in Table 4, our model excels in property prediction tasks compared to the relevant baselines. In zero-shot scenarios, MolLM achieves a score of 72.4 while MoMu achieves 66.96 on average. MolLM outperforms MoMu by up to 8.14%, demonstrating enhanced generalization capabilities. Upon fine-tuning, MolLM’s performance surpasses that of the baselines by significant margins, evident in datasets such as TOX21, where it increases from 75.6% to 80.0%, and BACE, where it improves from 77.1% to 84.1%. These enhancements underscore the efficacy of our expressive molecular encoder, which captures important 3D information. This aspect becomes crucial for classification tasks where considering molecular structure is important.

**Table 4.** Results of the property prediction task compared with other relevant baselines that also utilize a multimodal biomedical text and molecule model.^a^

Dataset	BBBP	TOX21	ToxCast	SIDER	ClinTox	MUV	HIV	BACE	Average
Zero-shot									
MoMu	65.8 ± 4.5	74.0 ± 0.8	63.4 ± 0.6	57.3 ± 1.6	58.0 ± 4.4	71.8 ± 2.5	75.3 ± 1.9	70.1 ± 5.4	66.96
MolLM	**70.6 ± 1.2**	**77.5 ± 0.7**	**65.7 ± 1.3**	**67.1 ± 0.9**	**70.8 ± 1.1**	**74.5 ± 0.6**	**76.1 ± 1.4**	**76.9 ± 0.8**	72.40
Fine-tune									
Random Forest (RF)	71.4 ± 0.0	76.9 ± 1.5	–	68.4 ± 0.9	71.3 ± 5.6	63.2 ± 2.3	78.1 ± 0.6	86.7 ± 0.8	–
RXNFP	68.5 ± 0.7	65.5 ± 0.5	–	54.0 ± 1.6	–	–	73.5 ± 1.0	–	–
BERT	68.4 ± 0.5	68.8 ± 0.7	–	60.2 ± 1.2	–	–	69.4 ± 1.0	–	–
SMI-BERT	71.1 ± 2.2	69.6 ± 0.4	–	59.9 ± 0.9	–	–	73.9 ± 1.5	–	–
GCN	71.8 ± 0.9	70.9 ± 2.6	–	53.6 ± 3.2	62.5 ± 2.8	71.6 ± 4.0	74.0 ± 3.0	71.6 ± 2.0	–
GIN	65.8 ± 4.5	74.0 ± 0.8	–	57.3 ± 1.6	58.0 ± 4.4	71.8 ± 2.5	75.3 ± 1.9	70.1 ± 5.4	–
KPGT	90.8 ± 1.0	**84.8 ± 1.3**	**74.6 ± 0.2**	64.9 ± 0.9	94.6 ± 2.2	–	–	–	–
KANO	**96.0 ± 1.6**	83.7 ± 1.3	73.2 ± 1.6	65.2 ± 0.8	94.4 ± 0.3	**83.7 ± 2.3**	**85.1 ± 2.2**	**93.1 ± 2.1**	84.3
MoLFormer-XL	93.7	84.7	–	**69.0**	**94.8**	–	82.2	88.21	–
KV-PLM	72.0 ± 0.9	70.0 ± 0.5	55.0 ± 1.7	59.8 ± 1.5	89.2 ± 2.7	54.6 ± 4.8	71.8 ± 1.4	78.5 ± 2.7	68.73
MoMu-S	70.5 ± 2.0	75.6 ± 0.3	63.4 ± 0.5	60.5 ± 0.9	79.9 ± 4.1	70.5 ± 1.4	75.9 ± 0.8	76.7 ± 2.1	71.63
MoMu-K	70.1 ± 1.4	75.6 ± 0.5	63.0 ± 0.4	60.4 ± 0.8	77.4 ± 4.1	71.1 ± 2.7	76.2 ± 0.9	77.1 ± 1.4	71.36
MolLM	**75.7 ± 1.7**	80.0 ± 1.7	68.2 ± 0.4	71 ± 0.8	**91.1 ± 1.0**	**79 ± 1.2**	**80.2 ± 0.7**	**84.1 ± 0.9**	76.16
MolLM + RF	66.7 ± 5.5	**80.1 ± 1.8**	**88.6 ± 0.9**	**80.8 ± 4.5**	70.7 ± 1.9	74.3 ± 0.8	77.0 ± 0.7	70.6 ± 4.2	76.10

a We also include other fine-tuned models for comparison, but only directly compare against the aforementioned relevant baselines. "Bold value" refers to the best performance.

### 4.3 Molecule captioning

**Figure 6.**
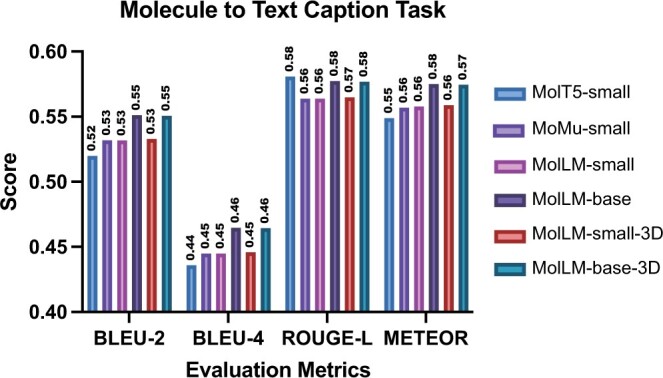
Our results for the molecule captioning task. We utilize the ChEBI dataset (Edwards *et al.* 2022) with 33 010 molecules. We use BLEU-2, BLEU-4, ROUGE-L, and METEOR to compare performance with MolT5 and MoMu as baselines. “-small” and “-base” suffixes on model names indicate the size of the MolT5 decoder because MolLM is encoder-only.

We utilize the molecule captioning task as outlined in (Edwards *et al.* 2022). In this task, our model generates text relating to a molecule based on its SMILES string, 2D graphs, and 3D graphs. For baseline comparisons, we select MolT5 (Edwards *et al.* 2022), which generates captions by inputting SMILES strings into the encoder-decoder transformer architecture. Another baseline we consider is MoMu (Su *et al.* 2022), which extends this process by appending 2D structural data to the SMILES embedding inputs. To improve our model’s understanding of the molecular structure and generate more accurate descriptions, we integrate 3D data into the input. Using the CheBI-20 dataset as in (Edwards *et al.* 2022), we compare the performance of MolLM, MoMu, and MolT5 using four different captioning metrics, as shown in Fig. 6. In summary, MolLM performs equivalently or slightly better than each baseline. This reveals that even without pre-training a text decoder, MolLM representations obtained through the robust encoder are sufficient for generating textual descriptions of molecules. This outcome underscores the versatility of our approach, its ready plug-and-play compatibility, and its ability to conserve computational resources. It can effortlessly leverage other models’ decoders to meet varying needs. Simultaneously, we acknowledge that this dataset has its limitations. Its small scale may not fully demonstrate the potential of our model.

### 4.4 Molecule editing

Following the procedure and settings outlined in (Liu *et al.* 2023b), we begin with 200 randomly sampled molecules from ZINC (Sterling and Irwin 2015) along with a brief text editing prompt as inputs. The task involves performing single-objective editing with prompts provided by Liu *et al.* such as “this molecule has high permeability.” These prompts direct the model to edit a molecule toward a specific molecular property. We evaluate each metric by its satisfactory “hit” ratio, which measures the ratio of the 200 molecules that the model can modify following the prompt, where a successful modification is defined as a “hit.” The determination of hits relies on calculated molecular metrics, each corresponding to a prompt. For example, a satisfactory hit for the “high permeability” prompt would involve the model outputting a molecule with increased permeability, measured by a lower calculated topological polar surface area. For other properties—hydrogen bond acceptors, hydrogen bond donors, drug-likeness, and solubility—the respective calculated properties used to determine hits are calculated hydrogen bond acceptors, calculated hydrogen bond donors, QED drug-likeness (Bickerton *et al.* 2012), and Wildman–Crippen LogP values (Wildman and Crippen 1999).

Upon examining the results for molecule editing, we noted distinct hit ratios for diverse properties. The model achieved a success ratio of 46.0% for increasing hydrogen bond acceptors and 54.5% for bolstering hydrogen bond donors. Yet, there was a clear dichotomy when modifying drug-likeness—the model attained a low hit ratio of 5.0% for rendering molecules more drug-like, compared to a considerably higher ratio of 81.5% for achieving the opposite. This discrepancy stems from the stark differences in the sizes of these two subtasks’ respective search spaces. In terms of editing for permeability, MolLM increases permeability with a success ratio of 33.5% and reduces it with an efficacy of 56.5%. This disparity could possibly be linked to the artificially set parameters used to define a satisfactory hit. Interestingly, the model performed well in enhancing solubility in water, achieving a hit ratio of 76.5%. In sum, these results suggest that while MolLM shows potential in certain molecular editing tasks, there is still room for refinement to bolster its performance. Refer to Supplementary appendix S9 for details on the magnitude of the errors made by our model during molecule editing.

## 5 Discussion

We selected a wide range of tasks to showcase MolLM’s flexibility in multiple contexts. For property prediction, our model does not explicitly use the text encoder. However, even without direct usage of text, the embeddings generated through molecule encoding are still influenced by the contrastive learning process, indirectly benefiting from biomedical text knowledge. This concept is evident in our utilization of fixed encoders for tasks such as employing MolT5 for molecule captioning and incorporating MoFlow for molecule generation. By maintaining our encoder in a fixed state during fine-tuning, we enable a better understanding of fine-tuning effects, prevent overfitting, and reduce computational costs. These tasks can perform well because the encoders are pre-trained and our multimodal approach provides sufficient context before fine-tuning.

Our results significantly surpassed current state-of-the-art baselines in cross-modality matching and property prediction tasks. In the matching task, incorporating 3D data yielded substantially better results compared to its 2D counterpart, with both outperforming all baselines. These outcomes not only underscore the importance of including multimodal contexts for pre-training but also highlight the particular significance of 3D encoding.

In molecule captioning, our model also achieved competitive results compared to baselines. Similar to the property prediction task, this task also only utilizes the molecular encoder of our model but still benefits from text knowledge via the contrastive learning process. We attribute these results primarily to the size of our dataset, which is larger than other text-based datasets. Interestingly, we observed that the inclusion of 3D data does not significantly improve results in this task. This discrepancy could be due to the heavily text-focused nature of the task, where the text may implicitly not require a large amount of molecule structure data. In other words, the captions themselves may not strongly correlate with molecular structure data. Conversely, captions such as the “molecule is symmetrical” may be more likely to benefit from incorporating molecular structural data.

In molecule editing, the performance varied significantly depending on the editing task. This variation could be due to the difficulty of specific prompts, as some prompts yielded successful hits for a much narrower range of similar molecules to the original. However, our model has constraints limiting the extent of molecule editing to avoid excessive alterations. For example, in the case of the solubility prompt, over-editing could potentially result in a highly soluble molecule that bears no resemblance to the original. Additionally, the variation in performance among the prompts might stem from inherent limitations within the training data, particularly in how frequently the text related to a specific prompt occurs.

Upon further investigation, we found that many edits resulted in molecules vastly different from the original, suggesting that a high hit ratio might also arise from overly editing a molecule. Therefore, we also report a similarity metric defined as the mean Tanimoto coefficient, ranging from 0.0 to 1.0, calculated between the RDKit molecular fingerprints of the original molecule and its modified version (Table 5). The similarity metrics consistently hover around 0.51, indicating that the model applies a fairly uniform level of modifications to the molecules, at least on average. This highlights issues with relying solely on the hit ratio metric for evaluation and suggests a potential need for developing a more robust metric. However, assessing the hit ratio metric alone overlooks the importance of similarity. A key aspect of molecule editing is retaining high similarity to the original, yet this metric encourages edits that strongly favor the prompt without considering the original structure. A better evaluation metric could involve a combination of both similarity to the original molecule and adherence to the prompt.

**Table 5.** Results of molecule editing task presented on different prompts with MolLM-3D.^a^

Prompt	Hit ratio	Similarity
This molecule has more hydrogen bond acceptors.	46.0	0.52
This molecule has more hydrogen bond donors.	54.5	0.51
This molecule is like a drug.	5.00	0.49
This molecule is not like a drug.	81.5	0.54
This molecule has high permeability.	33.5	0.51
This molecule has low permeability.	56.5	0.53
This molecule is soluble in water.	76.5	0.52
This molecule is insoluble in water.	21.5	0.50

a The satisfactory hit ratio results for our model’s performance on this task for the selected 200 molecules are scaled to a range of 0.0–100.0. The similarity metric is defined as the mean Tanimoto coefficient, ranging from 0.0 to 1.0, between the original molecule and its modified version.

The value of integrating 3D information cannot be overstated. Through a multimodal approach, we capture a richer representation of molecules, highlighting the interplay between their structural and spatial features. Additionally, the quality and size of the utilized datasets play a pivotal role in the performance of these models. Based on the demonstrated effectiveness of this architecture, molecular models should progress toward embracing a multimodal approach that incorporates 3D data.

## 6 Conclusion

MolLM, with its emphasis on including 3D positional data, exhibits robust performance across a variety of downstream tasks, including cross-modality matching, property prediction, captioning, and editing. This transformative approach, supported by ablation studies of 3D data within three of the downstream tasks, underscores the importance of incorporating a richer molecular representation, highlighting the nuanced interplay between structural and spatial features. Additionally, it emphasizes the important role of incorporating biomedical knowledge via textual inputs. Improved performance on tasks that did not directly use the text encoder demonstrates that incorporating this knowledge indirectly into molecular representations via contrastive learning validates this approach. In the future, this textual input could be further augmented using GPT to enrich the available data pool for pre-training. Refer to Supplementary appendix S10 for more ideas on potential future directions. Our model demonstrates a promising future for utilizing computational methods that incorporate the wealth of existing biomedical literature to advance complex tasks such as drug discovery that require a complex understanding that transcends multiple modalities.

## Supplementary Material

btae260_Supplementary_Data

## References

[btae260-B1] An X , Chen X, Yi D et al Representation of molecules for drug response prediction. *Briefings in Bioinformatics*. 2022 Jan;23(1):bbab393. 10.1093/bib/bbab393 PMC8769696 34571534

[btae260-B2] Bickerton GR , Paolini GV, Besnard J et al Quantifying the chemical beauty of drugs. Nat Chem 2012;4:90–8. 22270643 10.1038/nchem.1243 PMC3524573

[btae260-B3] Chen J , Zhang L. A survey and systematic assessment of computational methods for drug response prediction. Brief Bioinform 2021;22:232–46. 31927568 10.1093/bib/bbz164

[btae260-B4] Chilingaryan G , Tamoyan H, Tevosyan A et al Bartsmiles: Generative masked language models for molecular representations. *arXiv preprint arXiv:2211.16349*. 2022. 10.1021/acs.jcim.4c00512 39054761

[btae260-B5] Coley CW , Barzilay R, Green WH et al Convolutional embedding of attributed molecular graphs for physical property prediction. J Chem Inf Model 2017;57:1757–72. 28696688 10.1021/acs.jcim.6b00601

[btae260-B6] Devinyak O , Havrylyuk D, Lesyk R et al 3d-morse descriptors explained. J Mol Graph Model 2014;54:194–203. 25459771 10.1016/j.jmgm.2014.10.006

[btae260-B7] Devlin J , Chang M-W, Lee K et al BERT: Pre-training of Deep Bidirectional Transformers for Language Understanding. In: *Proceedings of NAACL-HLT* 2019 (pp. 4171-4186).

[btae260-B8] Edwards C , Lai T, Ros K et al Translation between molecules and natural language. In Goldberg Y, Kozareva Z, Zhang Y (eds.), *Proceedings of the 2022 Conference on Empirical Methods in Natural Language Processing*. Abu Dhabi, United Arab Emirates: Association for Computational Linguistics, 2022, 375–413.

[btae260-B9] Fang Y , Zhang Q, Zhang N et al Knowledge graph-enhanced molecular contrastive learning with functional prompt. Nat Mach Intell 2023;5:542–53.

[btae260-B10] Gu Y , Tinn R, Cheng H et al Domain-specific language model pretraining for biomedical natural language processing. ACM Trans Comput Healthcare 2021;3:1–23.

[btae260-B11] Hu W , Fey M, Zitnik M et al Open graph benchmark: datasets for machine learning on graphs. Adv Neural Inf Process Syst. 2020;33:22118–33.

[btae260-B12] Hwang D , Yang S, Kwon Y et al Comprehensive study on molecular supervised learning with graph neural networks. J Chem Inf Model 2020;60:5936–45. 33164522 10.1021/acs.jcim.0c00416

[btae260-B13] Jiang J , Wang R, Wei G-W et al Ggl-tox: geometric graph learning for toxicity prediction. J Chem Inf Model 2021;61:1691–700. 33719422 10.1021/acs.jcim.0c01294 PMC8155789

[btae260-B14] Kajita S , Ohba N, Jinnouchi R et al A universal 3d voxel descriptor for solid-state material informatics with deep convolutional neural networks. Sci Rep 2017;7:16991. 29209036 10.1038/s41598-017-17299-w PMC5717226

[btae260-B15] Kim S , Chen J, Cheng T et al 10.1093/nar/gkac956

[btae260-B16] Kipf TN , Welling M. Semi-Supervised Classification with Graph Convolutional Networks. In: *International Conference on Learning Representations* 2016.

[btae260-B17] Kuenzi BM , Park J, Fong SH et al Predicting drug response and synergy using a deep learning model of human cancer cells. Cancer Cell 2020;38:672–84.e6. 33096023 10.1016/j.ccell.2020.09.014 PMC7737474

[btae260-B18] Kuzminykh D , Polykovskiy D, Kadurin A et al 3d molecular representations based on the wave transform for convolutional neural networks. Mol Pharm 2018;15:4378–85. 29473756 10.1021/acs.molpharmaceut.7b01134

[btae260-B19] Landrum G. Rdkit documentation. *Release*. 2013;1(1-79):4.

[btae260-B20] Li H , Zhao D, Zeng J. KPGT: knowledge-guided pre-training of graph transformer for molecular property prediction. In: *Proceedings of the 28th ACM SIGKDD Conference on Knowledge Discovery and Data Mining* 2022 (pp. 857-867).

[btae260-B21] Li S , Zhou J, Xu T et al Geomgcl: Geometric graph contrastive learning for molecular property prediction. In: *Proceedings of the AAAI conference on artificial intelligence* 2022 Jun 28 (Vol. 36, No. 4, pp. 4541-4549).

[btae260-B22] Li Z , Jiang M, Wang S et al Deep learning methods for molecular representation and property prediction. *Drug Discovery Today*. 2022 Dec 1;27(12):103373. 10.1016/j.drudis.2022.103373 36167282

[btae260-B23] Liu S , Wang H, Liu W et al Pre-training Molecular Graph Representation with 3D Geometry. In: *International Conference on Learning Representations* 2021 Oct 6.

[btae260-B24] Liu J , Lei X, Zhang Y et al The prediction of molecular toxicity based on bigru and graphsage. Comput Biol Med 2023a;153:106524. 36623439 10.1016/j.compbiomed.2022.106524

[btae260-B25] Liu S , Nie W, Wang C et al Multi-modal molecule structure-text model for text-based retrieval and editing. Nat Mach Intell 2023b;5:1447–57.

[btae260-B26] Lo K , Wang LL, Neumann M et al S2ORC: The semantic scholar open research corpus. In: *Proceedings of the 58th Annual Meeting of the Association for Computational Linguistics*, Online. Association for Computational Linguistics, 2020, 4969–4983.

[btae260-B27] Luo R , Sun L, Xia Y et al Biogpt: generative pre-trained transformer for biomedical text generation and mining. Brief Bioinform 2022a;23:bbac409. 36156661 10.1093/bib/bbac409

[btae260-B28] Luo S , Chen T, Xu Y et al One transformer can understand both 2d & 3d molecular data. In: *The Eleventh International Conference on Learning Representations* 2022 Sep 29.

[btae260-B29] Miao Y , Ma H, Huang J et al Recent advances in toxicity prediction: applications of deep graph learning. Chem Res Toxicol 2023;36:1206–26. 37562046 10.1021/acs.chemrestox.2c00384

[btae260-B30] Pei Q , Wu L, Gao K et al Leveraging Biomolecule and Natural Language through Multi-Modal Learning: A Survey. *arXiv preprint arXiv:2403.01528*. 2024.

[btae260-B31] Pogány P , Arad N, Genway S et al De novo molecule design by translating from reduced graphs to smiles. J Chem Inf Model 2018;59:1136–46. 30525594 10.1021/acs.jcim.8b00626

[btae260-B32] Radford A , Narasimhan K, Salimans T et al Improving language understanding by generative pre-training. *OpenAI blog*, 2018. https://s3-us-west-2.amazonaws.com/openai-assets/research-covers/language-unsupervised/language_understanding_paper.pdf.

[btae260-B33] Raffel C , Shazeer N, Roberts A et al Exploring the limits of transfer learning with a unified text-to-text transformer. J Mach Learn Res. 2020;21:1–67.34305477

[btae260-B34] Rong Y , Bian Y, Xu T et al Self-supervised graph transformer on large-scale molecular data. Adv Neural Inf Process Syst. 2020;33:12559–71.

[btae260-B35] Ross J , Belgodere B, Chenthamarakshan V et al Large-scale chemical language representations capture molecular structure and properties. Nat Mach Intell. 2022;4:1256–64.

[btae260-B36] Scholkopf B , Burges CJC, Girosi F et al Comparing support vector machines with gaussian kernels to radial basis function classifiers. IEEE Trans Signal Process 1997;45:2758–65.

[btae260-B37] Singhal K , Azizi S, Tu T et al Large language models encode clinical knowledge. *Nature*. 2023;**620**(7972):172–80. 10.1038/s41586-023-06291-2 PMC10396962 37438534

[btae260-B38] Stärk H , Beaini D, Corso G et al 3d infomax improves gnns for molecular property prediction. In: *International Conference on Machine Learning* 2022 Jun 28 (pp. 20479-20502). PMLR.

[btae260-B39] Sterling T , Irwin JJ. Zinc 15—ligand discovery for everyone. J Chem Inf Model 2015;55:2324–37. 26479676 10.1021/acs.jcim.5b00559 PMC4658288

[btae260-B40] Su B , Du D, Yang Z et al A molecular multimodal foundation model associating molecule graphs with natural language. *arXiv preprint arXiv:2209.05481*. 2022.

[btae260-B41] Thomas N , Smidt T, Kearnes S et al Tensor field networks: Rotation-and translation-equivariant neural networks for 3d point clouds. a*rXiv preprint arXiv:1802.08219*. 2018.

[btae260-B42] Vaswani A , Shazeer N, Parmar N et al Attention is all you need. *Advances in Neural Information Processing Systems*. 2017;30.

[btae260-B43] Wang S , Guo Y, Wang Y et al Smiles-bert: Large scale unsupervised pre-training for molecular property prediction. In: *Proceedings of the 10th ACM International Conference on Bioinformatics, Computational Biology and Health Informatics*, BCB ’19. New York, NY, USA: Association for Computing Machinery, 2019, 429–436.

[btae260-B44] Wang Y , Magar R, Liang C et al Improving molecular contrastive learning via faulty negative mitigation and decomposed fragment contrast. J Chem Inf Model 2022a;62:2713–25. 35638560 10.1021/acs.jcim.2c00495

[btae260-B45] Wang Y , Wang J, Cao Z et al Molecular contrastive learning of representations via graph neural networks. Nat Mach Intell 2022b;4:279–87.

[btae260-B46] Wei J , Bosma M, Zhao VY et al Finetuned Language Models are Zero-Shot Learners. In: *International Conference on Learning Representations* 2021.

[btae260-B47] Weininger D. Smiles, a chemical language and information system. 1. Introduction to methodology and encoding rules. J Chem Inf Comput Sci 1988;28:31–6.

[btae260-B48] Wildman S , Crippen G. Prediction of physicochemical parameters by atomic contributions. J Chem Inf Comput Sci 1999;39:868–73.

[btae260-B49] Wu Z , Ramsundar B, Feinberg EN et al MoleculeNet: a benchmark for molecular machine learning. *Chemical Science*. 2018;**9**(2):513–30. 10.1039/c7sc02664a PMC5868307 29629118

[btae260-B50] Xia J , Zhu Y, Du Y et al A systematic survey of chemical pre-trained models. In: *Proceedings of the Thirty-Second International Joint Conference on Artificial Intelligence* 2023 Aug 19 (pp. 6787-6795).

[btae260-B51] Xu K , Hu W, Leskovec J et al How Powerful are Graph Neural Networks?. In: *International Conference on Learning Representations* 2018.

[btae260-B52] Yang K , Swanson K, Jin W et al Analyzing learned molecular representations for property prediction. J Chem Inf Model. 2019;59:3370–88. 31361484 10.1021/acs.jcim.9b00237 PMC6727618

[btae260-B53] Yu L , Su Y, Liu Y et al Review of unsupervised pretraining strategies for molecules representation. Brief Funct Genomics 2021;20:323–32. 34342611 10.1093/bfgp/elab036

[btae260-B54] Zang C , Wang F. Moflow: an invertible flow model for generating molecular graphs. In: *Proceedings of the 26th ACM SIGKDD international conference on knowledge discovery & data mining* 2020 Aug 23 (pp. 617-626).

[btae260-B55] Zeng Z , Yao Y, Liu Z et al A deep-learning system bridging molecule structure and biomedical text with comprehension comparable to human professionals. Nat Commun. 2022;13:862. 35165275 10.1038/s41467-022-28494-3 PMC8844428

[btae260-B56] Zhang Z , Liu Q, Wang H et al Motif-based graph self-supervised learning for molecular property prediction. Adv Neural Inf Process Syst. 2021;34:15870–82.

[btae260-B57] Zhu J , Xia Y, Qin T et al Dual-view molecular pre-training. In: *Proceedings of the 29th ACM SIGKDD Conference on Knowledge Discovery and Data Mining* 2023 Aug 6 (pp. 3615-3627).

[btae260-B58] Zhu J , Xia Y, Wu L et al Unified 2d and 3d pre-training of molecular representations. In: *Proceedings of the 28th ACM SIGKDD conference on knowledge discovery and data mining* 2022 Aug 14 (pp. 2626-2636).

[btae260-B59] Zuranski AM , Martinez Alvarado JI, Shields BJ et al Predicting reaction yields via supervised learning. Acc Chem Res. 2021;54:1856–65. 33788552 10.1021/acs.accounts.0c00770

