# Supplementary material for: MolLM: a unified language model for integrating biomedical text with 2D and 3D molecular representations
Source: Bioinformatics. 2024 Jun 28;40(Suppl 1):i357–68. doi: 10.1093/bioinformatics/btae260 (PMC11256921; doi:10.1093/bioinformatics/btae260)
Supplement: btae260_Supplementary_Data [file btae260_supplementary_data.pdf]

1 Related Work on Models for Specific Molecular Tasks

Molecular property prediction is one of the most widely researched molecular tasks due to its potential to expedite traditionally expensive and time-consuming wet lab experiments. Previous works have aimed to develop models that perform well in this prediction task (Wang *et al.*, 2022; Liu *et al.*, 2023b; Wen *et al.*, 2022; Ross *et al.*, 2022). For example, MolCLR (Wang *et al.*, 2022) performs contrastive learning via graph neural networks (GNNs) and data augmentation. However, it is not annotated with any text descriptions, limiting MolCLR's ability to perform molecular tasks involving text.

Concerning other models capable of downstream property prediction, the representation learning method described in (Zhu *et al.*, 2022) combines 2D and 3D graph data. This method encodes atomic coordinates and interatomic distances, subsequently merging them with atom representations through GNNs. Demonstrating a significant improvement over 2D-only methods on downstream property prediction tasks, this method underscores the importance of 3D representations and proposes an approach to incorporate 3D information into the pre-training process. However, as this model solely operates with molecular data without incorporating textual data as input, it lacks flexibility for more generalized tasks, many of which involve textual data.

Although molecular property prediction does not require the use of text, several works have proposed molecular tasks that necessitate a combined understanding of both molecules and text. For instance, MolT5 introduced the molecule captioning task, which involves generating meaningful textual descriptions of molecules, capturing their most important properties, structural elements, and applications (Edwards *et al.*, 2022). Furthermore, MoleculeSTM introduced a molecule editing task, wherein the input comprises a molecule and a prompt, such as "this molecule is more soluble," aiming for an output of a molecule similar to the original but aligned with the prompt (Liu *et al.*, 2023a).

2 Property Prediction Head

Below are the details of the prediction head we append to the base molecular encoder within our model to adapt to the property prediction task.

The main motivations behind the prediction head layer involve reducing features to learn the most important features for property prediction in the linear layers, ensuring a uniform distribution of outputs with the normalization layers for training stability, and considering dropout to avoid both underfitting and overfitting (Liu *et al.*, 2023c). We perform a grid search to obtain the final prediction head layer dimensions and hyperparameters.

- Linear layer: 768×512
- 1D batch normalization: 512 features
- Dropout: rate of 0.2
- Activation: ReLU
- Linear layer: 512×300
- Activation: ReLU
- Dropout: rate of 0.2
- Linear layer: $300 \times C$, where C represents the number of categories for the specific property prediction task.

3 Use of RDKit

We use RDKit to represent molecules from SMILES strings and obtain their graph structure prior to transforming their structures to our specific graph format with PyTorch Geometric. The motivation behind having an upper threshold of root-mean-square deviation after performing molecular augmentation is to ensure that the augmentation does not result in a molecule that is too semantically different for use in contrastive learning.

4 Molecule Augmentation Examples

We utilize four different molecule augmentations during our pre-training process: Node Dropping, Random Subwalk, Chemical Transformation, and Subgroup Removal. We chose these augmentations because they edit the molecules in a manner in which we believe the semantic meaning of the molecule is kept, but it is still augmented enough for the model to be more robust and explore the latent space. A more detailed explanation of each of these processes can be found in section 3.3.1. Figure 1 depicts five examples of how these augmentations transform an original molecule into its augmented versions. Let's look at a few examples. The first row shows the molecule betaine. We can observe the effect of node dropping, as one of the carbons has been dropped from the nitrogen. The random subwalk began on the carbon atom, which was connected to the oxygen atoms, and traversed through the graph. The chemical transformation added an amine group to one of the carbon atoms. Subgroup removal retained only one subgroup of the betaine, based on BRICS decomposition. Another molecule we can examine is in the fourth row: acetylphosphate. We can see that node dropping caused an oxygen to be dropped from the phosphorus atom. The random subwalk has taken a walk of the molecular graph, starting from the oxygen atom that is double-bonded to the phosphorus atom. The chemical transformation has introduced an amine group to an oxygen atom. Subgraph removal has left us with a random subgroup of the graph, as dictated by BRICS decomposition.

5 Training Implementation Details

5.1 Cross-modality Matching

We utilize a contrastive loss between the graph representations and text representations for fine-tuning. See Equation 1, where m is the margin controlling how far apart embeddings of negative pairs should be relative to positive pairs, and δ_{ij} is the Kronecker delta function.

$$\sum_{i=1}^{N}\sum_{j=1}^{N}\max\left(0, m + \cos(x_{\text{graph},i}, x_{\text{text},j}) - \delta_{ij}\cos(x_{\text{graph},i}, x_{\text{text},i})\right) \tag{1}$$

Specifically, we utilize $m = 0.2$ throughout all of our fine-tuning for this task. We fine-tune for 60 epochs with a batch size of 64 at a learning rate of $5 \cdot 10^{-5}$ for each subtask.

5.2 Property Prediction

For the fine-tuned tasks, which are all classification tasks, we utilize a Binary Cross-Entropy loss. See Equation 2, where N is the number of samples, $\hat{y}_i$ is the predicted value, and y_i is the target label.

$$-\frac{1}{N}\sum_{i=1}^{N}[y_i \cdot \log(\hat{y}_i) + (1 - y_i) \cdot \log(1 - \hat{y}_i)] \tag{2}$$

We fine-tune for 200 epochs with a batch size of 32 with a learning rate of 10^{-4}. See Appendix 2 for details on the linear prediction head for the classification task.

5.3 Molecule Caption

We utilize a Cross-Entropy Loss between the predicted captions and target captions. See Equation 3 for details on this loss, where $p(y_{ij}|x_i)$ is the model's probability for a token given the input concatenated SMILES string embedding and molecule embedding, x_i.

$$-\frac{1}{N}\sum_{i=1}^{N}\sum_{j=1}^{M_i}\log(p(y_{ij}|x_i)) \tag{3}$$

We fine-tune for 10 epochs with a batch size of 16 with a learning rate of 10^{-4}.

5.4 Molecule Editing

Finally, for the molecule editing task, we do not fine-tune our model, as the task involves *de novo* generation. Instead, we optimize the predicted molecule such that its embedding through MolLM is more aligned with the text prompt's embedding within the MolLM latent space. We utilize a mean squared error (MSE) between the predicted molecule embedding and the original molecule embedding to maintain similarity to the original molecule. See Equation 4 for the MSE loss, where z_{pred} is the predicted molecule embedding, z_{orig} is the original molecule embedding, and N is the number of samples.

$$\frac{1}{N}\sum_{i=1}^{N}\left(z_{\text{pred},i} - z_{\text{orig},i}\right)^2 \tag{4}$$

Additionally, we incorporate a Dot Product-based loss to ensure alignment between the predicted molecule's embedding and the text prompt's embedding. See Equation 5 for the Dot Product-based loss, where z_{text} is the text prompt's embedding.

$$-\frac{1}{N}\sum_{i=1}^{N}\left(z_{\text{pred},i} \cdot z_{\text{text},i}\right) \tag{5}$$

This dual approach, with a linear combination of these two losses for similarity retention and alignment to the prompt, ensures that the edited molecules are related to the original while being edited in a manner relevant to the prompt.

6 Embedding Translation MLP

The weights of this MLP for projection from MoFlow latent space to MolLM latent space were trained to minimize cosine similarity between the translated embeddings and the actual MolLM embedding for molecules within the ZINC (Sterling and Irwin, 2015) dataset. The cosine similarity loss for N pairs, where z_{pred} is the predicted translation and z_{true} is the true embedding through MolLM's molecule encoder, is

$$-\frac{1}{N}\sum_{i=1}^{N}\frac{z_{pred} \cdot z_{true}}{\|z_{pred}\|\,\|z_{true}\|}. \tag{6}$$

7 Model Architecture Explanation

Our choice of model architecture was based on our selection of molecular representations and the advantages offered by Transformers in encoding molecules. For molecular representation, we examined prior works to inform our approach. While SMILES and SELFIES strings are simple and directly leverage natural language pipelines, previous literature has highlighted limitations in these representations. For instance, SMILES strings can represent the same molecule in multiple ways, leading to unnecessary data redundancy. Additionally, both SMILES and SELFIES representations lack the ability to encode 2D/3D spatial information, which leads to decreased performance in certain tasks.

Thus, we chose to focus on graph-based molecular representations. Our reasoning for continuing to use Transformers to encode molecules stems from the architecture's superior capability to handle sequences. In contrast to traditional GNN or graph isomorphism network (GIN) models, which primarily emphasize the graph structure of molecules, Transformers allow for more flexible encoding. The attention mechanism within Transformers captures molecular patterns, while the encoder-decoder structure allows us to easily leverage multimodal inputs for pre-training. Consequently, we can effectively utilize textual data as inputs, enabling our model to understand domain-specific knowledge associated with a given molecule.

8 Property Prediction Baseline Models

We briefly describe the method of the models that we compare against the property prediction task. Random Forest (RF) utilizes ensembles of decision trees (Ho, 1995). For this task, Zhu *et al.* (2021) uses molecular fingerprints, including key chemical properties and structural features, as input features for RF. They do not specify their hyperparameters for RF.

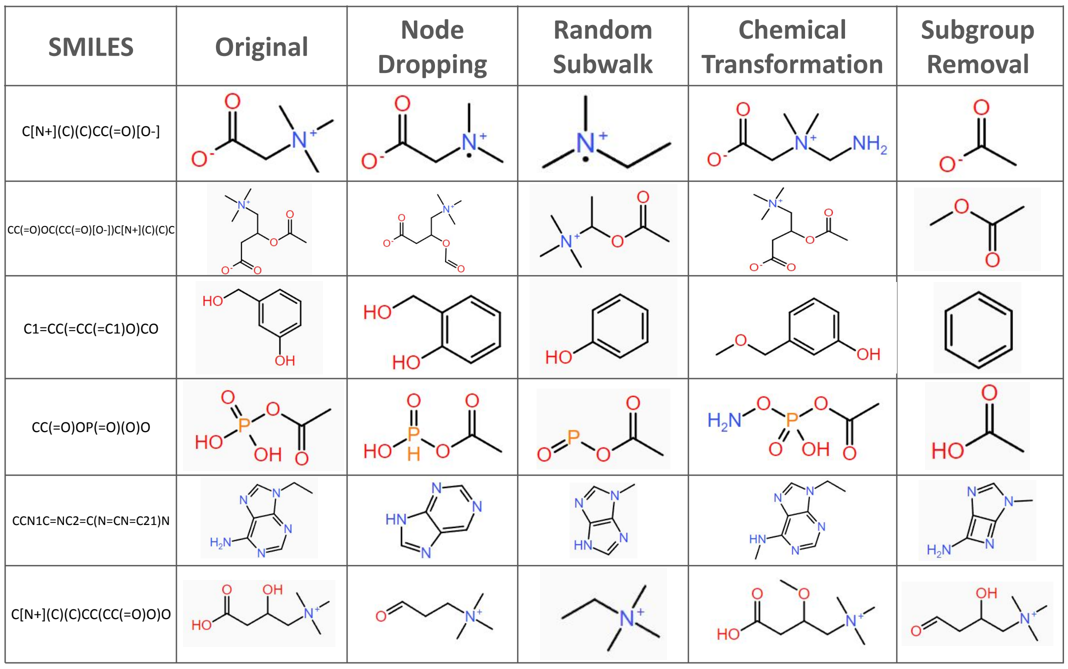

Fig. 1: Examples of different molecule augmentations. For each molecule, their SMILES string, original molecular graph, and the molecular graph of each of the four augmentations are shown.

RXNFP is a Transformer-based model trained on chemical reactions given as text (Schwaller *et al.*, 2021). Zeng *et al.* (2022) utilizes BERT (Devlin *et al.*, 2018) without pre-training. SMI-BERT is a BERT model pre-trained only on SMILES strings (Zeng *et al.*, 2022). Graph convolutional networks (GCNs) use convolution-like neighborhood aggregations to learn graph node and edge features (Kipf and Welling, 2016), which can be applied to molecular graphs. GINs utilize a more powerful learnable aggregation function than GCNs for enhanced expressivity (Xu *et al.*, 2018). KPGT introduces a Transformer model that utilizes line graphs generated from molecular graphs and additional knowledge from RDKit fingerprints (Li *et al.*, 2022). KANO integrates a chemical element knowledge graph into graph augmentations and utilizes a communicative message passing neural network architecture, which employs a directional approach to message passing between nodes (atoms) and a more dynamic approach to updating edge representations than traditional GNNs (Fang *et al.*, 2023). MoLFormer-XL utilizes a Transformer model with rotary positional embeddings and linear attention on SMILES strings (Ross *et al.*, 2022).

9 Molecule Editing Error Magnitude

We provide Figure 2 with the distribution of the measured respective metric across the generated molecules for each molecule editing prompt. We can not directly compare to MoleculeSTM at the time of writing this manuscript due to issues with their fine-tuned checkpoints and the fact that their output is not publicly available. Generally, the molecules that are edited in the opposite of the desired direction for the metric are clustered around no change (0 change in the metric). This indicates that most of the unsuccessfully edited molecules generally do not move the metric value too far in the wrong direction. For the worst-performing prompts, we attribute these to the innate complexity of the property and the frequency of its appearance in the training text corpus. For instance, changing an arbitrary molecule into a drug-like molecule is a significantly more difficult task than increasing its solubility where a small edit such as swapping one functional group may be sufficient.

Through the error analysis conducted by our recruited professionals, we provide an exploration of the worst results of our molecular edits in response to given prompts, including more hydrogen bond acceptors, hydrogen acceptors, increased drug likeness, and high permeability. Despite our model's efforts to modify the structures of molecules in response to the given prompts, the results proved unsuccessful across three instances (depicted in Figures 3, 4, and 5). In the case aiming for enhanced hydrogen bond acceptor capacity (Figure 3), the model removed the sulfonamide group and nitrogen within a thiocyanate-like structure, reducing the hydrogen bond accepting ability and hence contradicting the intended goal. The attempt to increase drug-likeness (Figure 4) led to the introduction of a triazine-like structure and a chlorinated diazene. Both modifications are less favorable due to potential stability and reactivity concerns, resulting in a decreased quantitative estimate of drug-likeness (QED). Finally, the endeavor to improve molecule permeability (Figure 5) resulted in increased polarity through the introduction of multiple amine and carboxyl groups. The increase in polarity inadvertently made the molecule more soluble in water but less able to cross lipid membranes via passive diffusion, thereby diminishing the molecule's permeability. These examples demonstrate the challenges that can arise for sophisticated molecular edits, which require a nuanced understanding of the interplay between different molecular properties.

10 Future Direction

In terms of future directions, there is an evident need for more extensive and better-curated datasets. Having larger datasets with a stronger correlation between text and molecular representations would enable the model to establish clearer distinctions among molecules. Automatically identifying such text in large text corpora poses challenges because the mention of molecules in certain texts does not guarantee their relevance. Thus, an improvement in the current text sampling method is necessary. For example, future studies could leverage language models to sift through academic texts or include additional quantitative data from other sources.

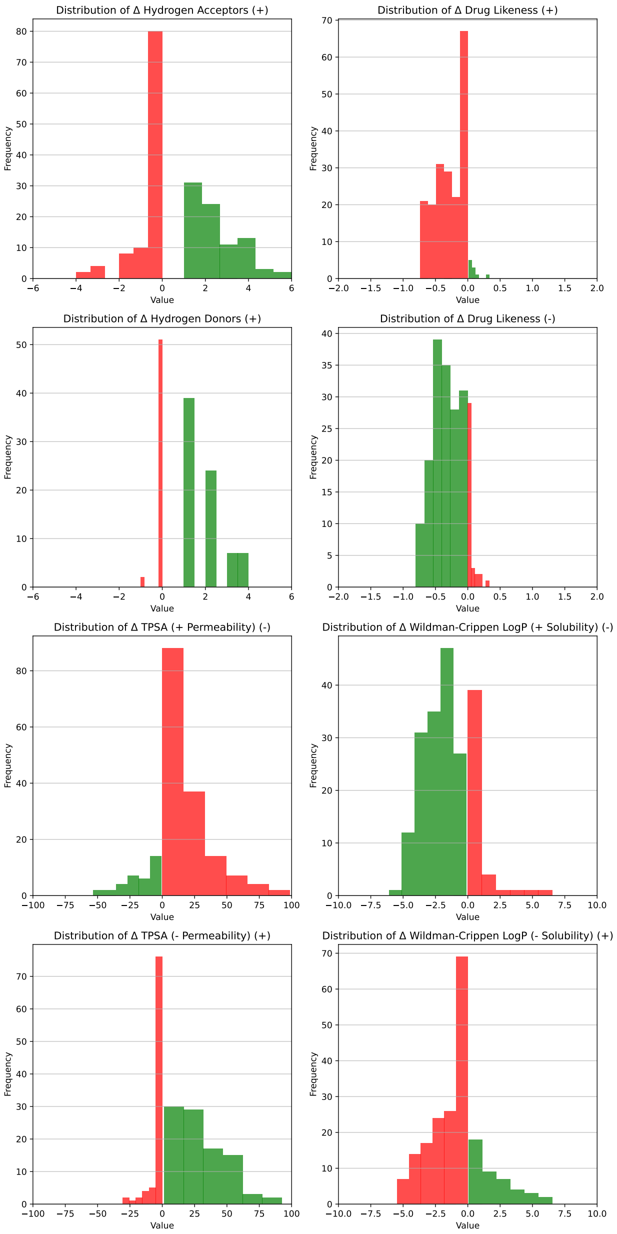

Fig. 2: Plots of the distribution of the measured metrics for the generated molecules from each instance of the editing task. The (+) and (-) at the end of each plot title indicate whether a higher or lower value of the metric is desired. Green indicates the set of molecules that move the metric in the desired direction while red represents those that do not. We additionally clarify that the desired direction change of the metric is opposite that of the phrasing of the prompt for both permeability and solubility. For instance, a lower TPSA value is desired when prompting for "higher permeability."

Exploring improvements in downstream tasks is also important. Introducing novel downstream tasks could allow us to validate the model's applicability across a broader spectrum of challenges in molecular biology and chemistry. Additionally, there exist numerous potential directions for improving the downstream tasks utilized by MolLM. For example, a major weakness of the molecule generation task lies in the fact that it sometimes generates infeasible molecules. Future works could aim to validate these generated molecules for chemical validity, chemical stability, or feasibility of synthesis. This could be achieved by employing techniques such as reinforcement learning from human feedback (Ziegler *et al.*, 2019; Stiennon *et al.*, 2020), creating a reward function based on expert human knowledge to fine-tune the generation model. Leveraging transfer learning from models like GPT-4 could also aid in this improvement.

**Prompt**	**This molecule has more hydrogen bond acceptors.**
Original Molecule	Cc1onc(-c2c(F)cccc2Cl)c1C(=O)Nc1ccc(S(=O)(=O)Nc2nccs2)cc1 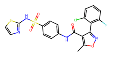
Edited Molecule	C[n+]1occ(C(=CF)N(Cl)CCBr)c1C(N)=O 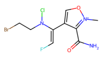
Δ Metric	-4 hydrogen acceptors
Discussion	The sulfonamide group, S(=O)(=O), and nitrogen within the thiocyanate-like structure are removed which reduces hydrogen acceptor capability. The nitrogen now carries a positive charge [n+] which makes it less available for hydrogen bonding. The addition of Br is not helpful as it is not typically a strong hydrogen bond acceptor due to its lower electronegativity.

Fig. 3: An Example of an unsuccessful molecule edit with its prompts, original molecule, edited molecule, and a discussion of the failures in the edit.

**Prompt**	**This molecule is like a drug.**
Original Molecule	O=C(NC[C@H]1CCCO1)c1ccccc1N1CCCC1=O 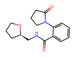
Edited Molecule	N=C1CCCN1N(NNCl)C(=N)C(=O)CCN1CCCO1 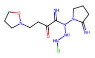
Δ Metric	-0.691 QED
Discussion	Amide groups, C(=O)N and N1CCCC1=O, are replaced by a triazine-like structure, N=C1CCCN1, and a chlorinated diazene, NNCl, both of which are less favorable for drug likeness due to potential stability and reactivity concerns. Making triazine-like ring N=C1CCCN1 may add rigidity which harms drug likeness.

Fig. 4: An Example of an unsuccessful molecule edit with its prompts, original molecule, edited molecule, and a discussion of the failures in the edit.

For our molecule editing task, future work could explore evaluating how well MoleculeSTM, our model, and other similar models allow for dynamic adjustment of the weighting between emphasizing retaining original molecular features and chemical changes that favor the given prompt. The motivation behind this is that there is likely often the case where a molecule already has many desirable properties and minimal editing is wanted to achieve a prompt. Moreover, a better metric could even consider the ratio of change in the molecule in favor of the prompt to how much the molecule is chemically altered. Our experiments also shed light on the lack of reliable baselines, especially in *de novo* generation, where there is no gold standard. This poses questions about the veracity and stability of generated molecules and presents the opportunity for the creation and adoption of better baselines.

Additionally, the editing task highlights the usefulness of incorporating natural language prompts as input into the model for powerful biomedical-related tasks. Exploring methods to enhance the robustness of this editing task, experimenting with various prompts, and proposing new tasks that involve natural language prompts could lead to more powerful tools leveraging the utility and ease of using natural language prompts.

Furthermore, the pre-training technique could be expanded upon in a manner more advanced than just curating larger or higher-quality datasets. There is also an avenue to explore utilizing much larger language models, such as ChatGPT (Jahan *et al.*, 2023) or LLaMA, as agents for biomedical tasks.

Finally, exploring different molecular encoding methods could yield promising results. For example, while Transformer-M combines 2D/3D data linearly, future works could experiment with non-linear combinations of 2D and 3D data. This approach could enhance expressiveness, enabling the model to better discern the nuances between 2D and 3D representations.

**Prompt**	**This molecule has high permeability.**
Original Molecule	CC1CCN(S(=O)(=O)c2ccc(C(=O)Nc3cc(Cl)ccc3Cl)cc2)CC1 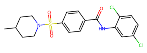
Edited Molecule	NC(=COOCl)N=CNN(N)N1C=CC(C(O)(O)N2CCC(O)CC2)=CC1 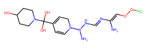
Δ Metric	+98.820 TPSA
Discussion	In the edited molecule, multiple amine groups (N=CNN(N)N) and carboxyl groups (C(O)(O)) are introduced. In this context, they increase the polarity of the molecule. Higher polarity means that the molecule is more water-soluble but less likely to cross lipid membranes by passive diffusion. This counteracts the intention to increase the permeability of the molecule.

Fig. 5: An Example of an unsuccessful molecule edit for high permeability with its prompts, original molecule, edited molecule, and a discussion of the failures in the edit.

References

Devlin, J. *et al.* (2018). Bert: Pre-training of deep bidirectional transformers for language understanding.

Edwards, C. *et al.* (2022). Translation between molecules and natural language. In Y. Goldberg, Z. Kozareva, and Y. Zhang, editors, *Proceedings of the 2022 Conference on Empirical Methods in Natural Language Processing*, pages 375–413, Abu Dhabi, United Arab Emirates. Association for Computational Linguistics.

Fang, Y. *et al.* (2023). Knowledge graph-enhanced molecular contrastive learning with functional prompt. *Nature Machine Intelligence*, **5**(5), 542–553.

Ho, T. K. (1995). Random decision forests. In *Proceedings of 3rd International Conference on Document Analysis and Recognition*, volume 1, pages 278–282 vol.1.

Jahan, I. *et al.* (2023). Evaluation of ChatGPT on biomedical tasks: A zero-shot comparison with fine-tuned generative transformers. In D. Demner-fushman, S. Ananiadou, and K. Cohen, editors, *The 22nd Workshop on Biomedical Natural Language Processing and BioNLP Shared Tasks*, Toronto, Canada. Association for Computational Linguistics.

Kipf, T. N. and Welling, M. (2016). Semi-supervised classification with graph convolutional networks.

Li, H. *et al.* (2022). Kpgt: Knowledge-guided pre-training of graph transformer for molecular property prediction.

Liu, S. *et al.* (2023a). Multi-modal molecule structure-text model for text-based retrieval and editing. *Nature Machine Intelligence*, **5**(12), 1447–1457.

Liu, Y. *et al.* (2023b). Molrope-bert: An enhanced molecular representation with rotary position embedding for molecular property prediction. *Journal of Molecular Graphics and Modelling*, **118**, 108344.

Liu, Z. *et al.* (2023c). Dropout reduces underfitting. In A. Krause, E. Brunskill, K. Cho, B. Engelhardt, S. Sabato, and J. Scarlett, editors, *Proceedings of the 40th International Conference on Machine Learning*, volume 202 of *Proceedings of Machine Learning Research*, pages 22233–22248. PMLR.

Ross, J. *et al.* (2022). Large-scale chemical language representations capture molecular structure and properties. *Nature Machine Intelligence*, **4**(12), 1256–1264.

Schwaller, P. *et al.* (2021). Mapping the space of chemical reactions using attention-based neural networks. *Nature Machine Intelligence*, **3**(2), 144–152.

Sterling, T. and Irwin, J. J. (2015). Zinc 15 – ligand discovery for everyone. *Journal of Chemical Information and Modeling*, **55**(11), 2324–2337.

Stiennon, N. *et al.* (2020). Learning to summarize with human feedback. *Advances in Neural Information Processing Systems*, **33**, 3008–3021.

Wang, Y. *et al.* (2022). Molecular contrastive learning of representations via graph neural networks. *Nature Machine Intelligence*.

Wen, N. *et al.* (2022). A fingerprints based molecular property prediction method using the bert model. *Journal of Cheminformatics*, **14**(1), 1–13.

Xu, K. *et al.* (2018). How powerful are graph neural networks?

Zeng, Z. *et al.* (2022). A deep-learning system bridging molecule structure and biomedical text with comprehension comparable to human professionals. *Nature communications*, **13**(1), 862.

Zhu, J. *et al.* (2021). Dual-view molecule pre-training.

Zhu, J. *et al.* (2022). Unified 2d and 3d pre-training of molecular representations.

Ziegler, D. M. *et al.* (2019). Fine-tuning language models from human preferences. *arXiv preprint arXiv:1909.08593*.
